# Low uptake of palliative care for COPD patients within primary care in the UK

**DOI:** 10.1183/13993003.01879-2017

**Published:** 2018-02-15

**Authors:** Chloe I. Bloom, Bhavan Slaich, Daniel R. Morales, Liam Smeeth, Patrick Stone, Jennifer K. Quint

**Affiliations:** 1 Respiratory Epidemiology, Occupational Medicine and Public Health, National Heart and Lung Institute, Imperial College London, London, UK; 2 Division of Population Health Sciences, University of Dundee, Dundee, UK; 3 Faculty of Epidemiology and Public Health, London School of Hygiene and Tropical Medicine, London, UK; 4 Marie Curie Palliative Care Research Unit, University College London, London, UK

## Abstract

Mortality and symptom burden from chronic obstructive pulmonary disease (COPD) and lung cancer are similar but there is thought to be an inequality in palliative care support (PCS) between diseases. This nationally representative study assessed PCS for COPD patients within primary care in the UK.

This was a cohort study using electronic healthcare records (2004–2015). Factors associated with receiving PCS were assessed using logistic regression for the whole cohort and deceased patients.

There were 92 365 eligible COPD patients, of which 26 135 died. Only 7.8% of the whole cohort and 21.4% of deceased patients received PCS. Lung cancer had a strong association with PCS compared with other patient characteristics, including Global Initiative for Chronic Obstructive Lung Disease stage and Medical Research Council Dyspnoea score (whole cohort, lung cancer: OR 14.1, 95% CI 13.1–15; deceased patients, lung cancer: OR 6.5, 95% CI 6–7). Only 16.7% of deceased COPD patients without lung cancer received PCS compared with 56.5% of deceased patients with lung cancer. In patients that received PCS, lung cancer co-diagnosis significantly increased the chances of receiving PCS before the last month of life (1–6 *versus* ≤1 month pre-death: risk ratio 1.4, 95% CI 1.3–1.7).

Provision of PCS for COPD patients in the UK is inadequate. Lung cancer, not COPD, was the dominant driver for COPD patients to receive PCS.

## Abstract 2

**Palliative care provision for COPD patients without lung cancer is poor in the UK** http://ow.ly/IIC230gWOOV

## Introduction

Globally, chronic obstructive pulmonary disease (COPD) prevalence is on the rise and is the only disease that continues to have an increasing age-adjusted mortality rate [1]. In the UK, 5.2% of all deaths are secondary to COPD, which is approaching the proportion (6.2%) of deaths that are due to lung cancer [2, 3]. Patients with advanced COPD have a burden of disabling physical symptoms that are often compounded by multiple comorbidities, psychological distress and social isolation. Currently, there is a lack of palliative care support (PCS) for patients with end-stage COPD, despite evidence that it improves their quality of life [4, 5]. The natural course of physical decline for patients with COPD can be variable, but overall it is characterised by a long-term steady deterioration [6]. However, most healthcare resources are dedicated towards management and prevention of acute events, with significantly less emphasis from physicians and researchers on palliative and supportive care [7].

The 2010 National Institute for Health and Clinical Excellence (NICE) guidelines state that patients with end-stage COPD should have access to the full range of services offered by palliative care teams [8]. These include palliation for breathlessness and other symptoms, advance care planning, addressing emotional and social needs, and end-of-life care. However, compared with patients with lung cancer who suffer from a similar burden of disabling symptoms and psychological distress, small studies or studies using select COPD cohorts have found over the past decade that COPD patients often have limited access to palliative care services [9–13], even when compared with patients with other chronic terminal diseases [14]. It is likely that a major reason for this is the difficulty in predicting when to initiate PCS; current recommendations are to deliver PCS during the last year of life, but prognosticating the life expectancy of individuals with COPD has proven to be extremely difficult [1, 15, 16]. Consequentially, there is no commonly accepted definition of “end-stage COPD”. In addition, it is well recognised that communication between COPD patients with advanced disease and physicians remains poor regarding disease prognosis and end-of-life planning, which is likely to be related to a lack of training and guidance [17, 18].

Previous studies have investigated the planning of PCS from acute UK hospital trusts; unfortunately, these have consistently found poor access for COPD patients to palliative care services, alongside limited provision of patient information [19, 20]. This is the first nationally representative study to describe the uptake of PCS in COPD patients, the characteristics that are associated with receiving PCS and how this has changed over the past decade.

## Methods

### Data sources

The UK Clinical Practice Research Datalink (CPRD) includes 674 general practitioner (GP) practices and has current coverage of over 11.3 million patients, and represents the UK's population with respect of age, sex, body mass index (BMI) and ethnicity [21]. Approximately 60% of CPRD practices have patient-level linkage to Hospital Episode Statistics (HES) data and Office of National Statistics (ONS) mortality data.

### Study design and population

**FIGURE 1:**
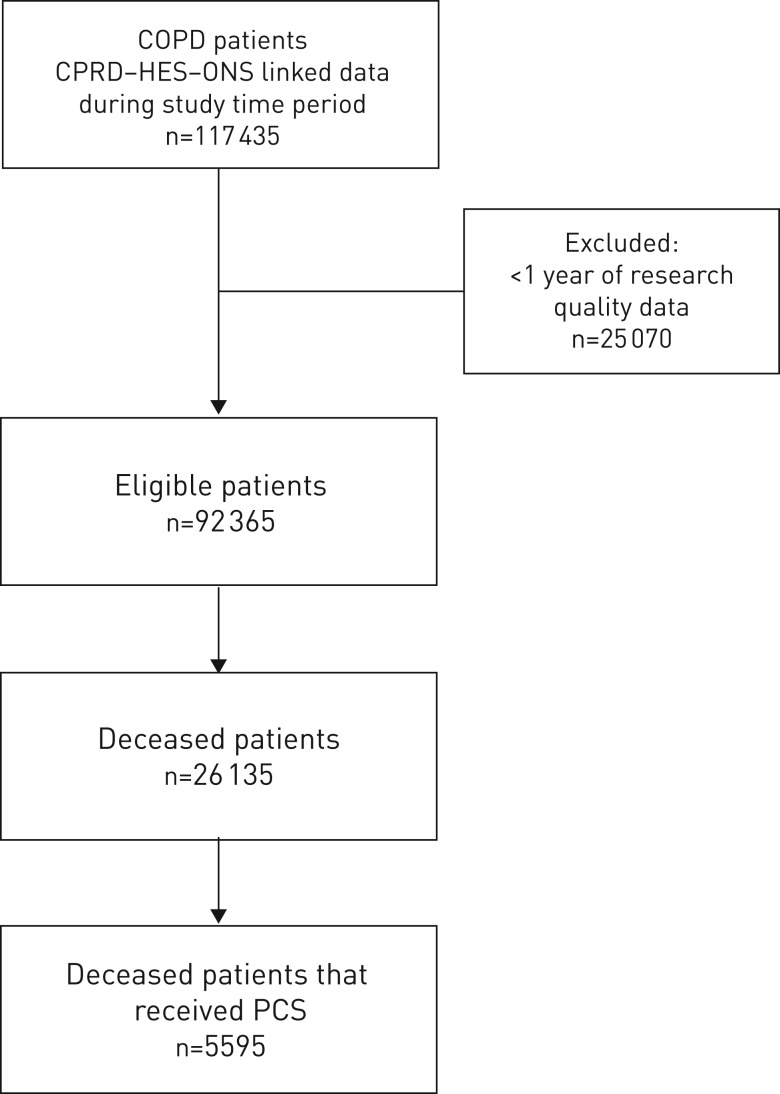
Flowchart of patient inclusion in the study. COPD: chronic obstructive pulmonary disease; CPRD: Clinical Practice Research Datalink; HES: Hospital Episode Statistics; ONS: Office of National Statistics; PCS: palliative care support.

We conducted an open cohort study of COPD patients that contribute towards routinely collected CPRD–HES–ONS linked electronic healthcare records (figure 1). Patients had to have a COPD diagnosis as determined using a previous validated algorithm [22]. Patients entered the study 1 year after their latest COPD diagnosis date, the date the practice began recording research quality data, their continuous CPRD-GP registration date or study start date (December 31, 2004). Patients were censored at date of death, the end of the study period (March 31, 2015), the GP practice last data collection date or the date of transfer out of a CPRD-linked practice.

### Outcome and variables

The primary outcome was documentation of PCS, as recorded in the patients' electronic healthcare records using relevant Read Codes (the clinical terminology system used by CPRD); two-thirds of codes were for “terminal care”, “palliative care”, “on gold standards palliative care framework” or “palliative care plan review”; other commonly used codes included “palliative care treatment”, “palliative care specialist” or “seen by palliative care physician” (supplementary table S1). A history of smoking (current or ex-smoker), myocardial infarction, lung cancer, heart failure, stroke, anxiety and depression was identified using appropriate Read Codes. BMI (kg·m^−2^) was categorised as underweight (<19 kg·m^−2^), normal (19–25 kg·m^−2^), overweight (25–30 kg·m^−2^) or obese (≥30 kg·m^−2^). Exacerbations were identified using a validated algorithm [23]; the baseline year was defined as the year before study entry. A moderate exacerbation was defined as having been treated within the GP practice, while a severe exacerbation was defined as requiring hospitalisation; exacerbations recorded within 14 days of the index exacerbation were considered as the same event. COPD severity was classified using Global Initiative for Chronic Obstructive Lung Disease (GOLD) staging classification and the Medical Research Council (MRC) Dyspnoea scale [24, 25]. Death was defined as death from any cause in the analysis; however, in a sensitivity analysis, we investigated a subgroup of patients for whom it was highly likely COPD led directly to the cause of death based upon ONS data.

### Statistical analysis

Logistic regression was used to calculate the association between patient characteristics and PCS, for the whole cohort and for the deceased patients. Multivariate logistic regression was conducted using the following variables: sex, age at study entry, history of myocardial infarction, stroke, lung cancer, anxiety, depression, heart failure, BMI, GOLD stage, MRC Dyspnoea score, smoking status and number of exacerbations in the baseline year. These demographic and clinical variables, taking into account disease severity and comorbidity, were selected as known or suspected risk factors for receiving PCS [11–13]. Where data was <25% missing, a complete case analysis was undertaken; for variables with >25% missing, the variable was excluded from the multivariate regression model (GOLD stage in the whole cohort and deceased subpopulation, and MRC Dyspnoea score in the deceased subpopulation) and a sensitivity analysis was then carried out to include that variable in the model. Multinomial regression was applied to estimate the relative effect of a coexisting lung cancer diagnosis on the timing of the PCS in relation to time of death. Two time periods, *i.e.* “early period” (6–12 months before death) and “medium period” (1–6 months before death), were compared to the “late period” (≤1 month before death). Statistical analysis was conducted using Stata version 14.1 (StataCorp, College Station, TX, USA).

### Ethics

The protocol for this research was approved by the Independent Scientific Advisory Committee (ISAC) for Medicines and Healthcare Products Regulatory Agency Database Research (protocol 17_083); the approved protocol was made available during peer review. Generic ethical approval for observational research using the CPRD with approval from the ISAC was granted by a Health Research Authority Research Ethics Committee (East Midlands – Derby; 05/MRE04/87).

## Results

### Characteristics of the whole cohort

There were 92 365 eligible COPD patients in the cohort (figure 1). At study entry, mean age was 67.8 years, 49 801 (53.9%) were male, 18 962 (20.6%) had cardiovascular disease (myocardial infarction, stroke or heart failure), 31 260 (33.8%) had anxiety or depression, 30 656 (34.1%) had a normal BMI, 46 900 (52.3%) were current smokers, 19 841 (35.2%) had GOLD stage ≥3, 18 295 (25.5%) had MRC Dyspnoea score ≥4, 46 898 (50.8%) had no exacerbations in their baseline year and 26 135 (28.3%) died during study follow-up (table 1).

**TABLE 1:** Demographic and clinical characteristics of the whole chronic obstructive pulmonary disease (COPD) cohort and the deceased COPD patients from 2005 to 2014

	**Whole cohort**			**Deceased patients**		
	**Whole cohort**	**Received PCS**	**No PCS**	**Deceased patients**	**Received PCS**	**No PCS**
**Total (% of total)**	n=92 365 (100)	n=7198 (7.8)	n=85 167 (92.2)	n=26 135 (100)	n=5595 (21.4)	n=20 540 (78.6)
**Follow-up years**	4.2 (1.9–7.2)	4.3 (2.2–7.0)	4.1 (1.9–7.3)	3.3 (1.5–5.6)	3.9 (2–6.3)	3.1 (1.4–5.3)
**Age years**	67.8±11.4	71.8±9.7	67.5±11.6	74±9.6	72.3±9.3	74.5±9.5
**Age category years**						
≤65	35 994 (39)	1703 (23.7)	34 291 (40.3)	4628 (17.7)	3408 (16.6)	1220 (21.8)
65–75	29 424 (31.9)	2624 (36.5)	26 800 (31.5)	8339 (31.9)	6296 (30.7)	2043 (36.5)
≥75	26 947 (29.2)	2871 (39.9)	24 076 (28.3)	13 168 (50.4)	10 836 (52.8)	2332 (41.7)
**Male**	49 801 (53.9)	4134 (57.4)	45 667 (53.6)	15 215 (58.2)	3256 (58.2)	11 959 (58.2)
**Myocardial infarction**	6232 (7.4)	726 (10.1)	6309 (7.4)	3004 (11.5)	576 (10.3)	2428 (11.8)
**Stroke**	5526 (6.1)	573 (8)	5062 (5.9)	2390 (9.1)	451 (8.1)	1939 (9.4)
**Heart failure**	9673 (10.6)	1140 (15.8)	8747 (10.3)	5335 (20.4)	903 (16.1)	4432 (21.6)
**Lung cancer**	4138 (4.5)	2071 (28.8)	2380 (2.8)	3121 (11.9)	1764 (31.5)	1357 (6.6)
**Anxiety**	19 990 (21.9)	1765 (24.5)	18 536 (21.8)	5425 (20.8)	1356 (24.2)	4069 (19.8)
**Depression**	21 836 (23.9)	1693 (23.5)	20 459 (24.0)	5375 (20.6)	1268 (22.7)	4107 (20)
**BMI kg·m**^−^**^2^**	n=86 789	n=6 486	n=89 789	n=24 686	n=5382	n=19 304
<19 (underweight)	8144 (9.1)	1060 (15.3)	7084 (8.6)	4075 (16.5)	843 (15.7)	3232 (16.7)
19–25 (normal)	30 656 (34.1)	2780 (40)	27 876 (33.7)	9842 (39.9)	2207 (41)	7635 (39.6)
25–30 (overweight)	27 738 (30.9)	1854 (26.7)	25 884 (31.3)	6272 (25.4)	1417 (26.3)	4855 (25.2)
≥30 (obese)	23 251 (25.9)	1253 (18)	21 998 (26.7)	4497 (18.2)	915 (17)	3582 (18.6)
**Smoking status**	n=89 789	n=6726	n=84 392	n=24 686	n=5382	n=19 304
Current smoker	46 900 (52.3)	3440 (49.5)	43 460 (52.5)	11 744 (47.6)	2646 (49.2)	9098 (47.1)
Ex-smoker	42 889 (47.8)	3507 (50.5)	39 382 (47.5)	12 942 (52.4)	2736 (50.8)	10 206 (52.9)
**GOLD stage**	n=56 159	n=5017	n=59 362	n=16 118	n=3614	n=12 504
1	12 996 (23.1)	917 (19.7)	12 049 (23.4)	3411 (21.2)	723 (20)	2688 (21.5)
2	23 352 (41.6)	1651 (35.5)	21 701 (42.1)	4989 (31)	1249 (34.6)	3740 (29.9)
3	15 259 (27.2)	1442 (31)	13 817 (26.8)	5360 (33.3)	1132 (31.3)	4228 (33.8)
4	4582 (8.2)	643 (13.8)	3939 (7.7)	2358 (14.6)	510 (14.1)	1848 (14.8)
**MRC Dyspnoea score**	n=71 985	n=4656	n=66 177	n=15 104	n=3668	n=11 436
1	11 168 (15.5)	393 (7.7)	10 775 (16.1)	1156 (7.7)	296 (8.1)	860 (7.5)
2	23 856 (33.1)	1099 (21.6)	22 757 (34)	3097 (20.5)	774 (21.1)	2323 (20.3)
3	18 666 (25.9)	1233 (24.2)	17 433 (26)	3899 (25.8)	877 (23.9)	3 022 (26.4)
4	13 644 (19)	1421 (27.9)	12 223 (18.3)	4431 (29.3)	1011 (27.6)	3420 (29.9)
5	4651 (6.5)	943 (18.5)	3708 (5.5)	2521 (16.7)	710 (19.4)	1811 (15.8)
**Exacerbations in baseline year**	n=92 365	n=6726	n=91 201	n=26 135	n=5595	n=20 540
0	46 898 (50.8)	3227 (44.8)	43 671 (51.3)	11 762 (45)	2516 (45)	9246 (45)
≤2 (moderate)	27 991 (30.3)	2178 (30.3)	25 813 (30.3)	7690 (29.4)	1695 (30.3)	5995 (29.9)
≥3 moderate/≥1 severe	17 476 (18.9)	1793 (24.9)	15 683 (18.4)	6683 (25.6)	1384 (24.7)	5299 (25.8)

Data are presented as median (interquartile range), mean±sd or n (%), unless otherwise stated. PCS: palliative care support; BMI: body mass index; GOLD: Global Initiative for Obstructive Lung Disease; MRC: Medical Research Council.

During follow-up (median (interquartile range (IQR)) 4.2 (1.9–7.2) years), only 7198 (7.8%) received PCS through primary care. 4138 (4.5%) of the whole cohort were diagnosed with lung cancer by the end of follow-up. In patients without a co-diagnosis of lung cancer, only 5.8% (5127 out of 88 227) received PCS; in contrast, in the subgroup of patients with COPD and lung cancer, 50% (2071 out of 4138) received PCS.

### Characteristics of the deceased patients

26 135 patients died during the cohort follow-up. Compared with the whole cohort, deceased patients had a slightly shorter follow-up time (median (IQR) 3.3 (1.5–5.6) years), were slightly older (mean age 74 years), but had a similar percentage of males (table 1). The proportion of patients with a history of myocardial infarction, stroke, heart failure or lung cancer was higher in deceased COPD patients than the cohort as a whole; anxiety and depression were of similar proportions. There was a higher proportion of deceased COPD patients when compared with the whole cohort, who were underweight, ex-smokers, had a higher GOLD stage, higher MRC Dyspnoea scores and more exacerbations in the baseline year (table 1). During follow-up (median (IQR) 3.7 (1.5–5.6) years), only 4655 (22.5%) received or accessed PCS through primary care. In patients without a co-diagnosis of lung cancer, only 16.7% (3831 out of 23 014) received PCS; in contrast, in the subgroup of patients with COPD and lung cancer, 56.5% (1764 out of 3121) received PCS. In the sensitivity analysis, including only patients known to have died from their COPD, there were 15 745 patients, only 15.4% (1641) of whom had received PCS.

### Longitudinal changes in the proportion of patients receiving PCS from 2005 to 2014

**FIGURE 2:**
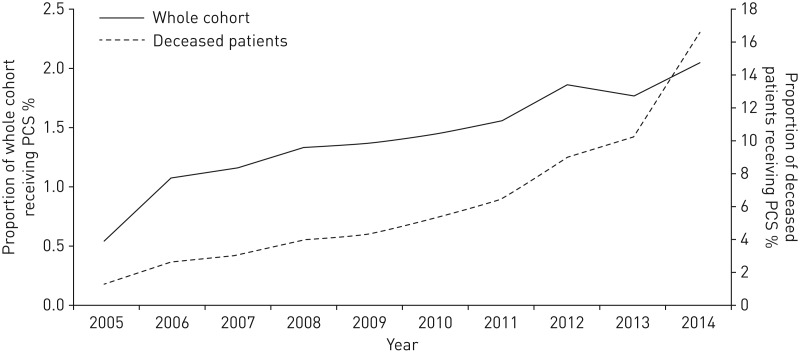
Proportion of chronic obstructive pulmonary disease patients that received palliative care support (PCS) in each year during study follow-up.

From 2005 to 2014, there was a steady rise in the proportion of patients who received PCS (figure 2). In 2005, 0.5% (216 out of the 39 685 patients in the cohort in 2005) had received support; by 2014, 2.0% (907 out of the 44 493 patients in the cohort in 2014) had received PCS. The proportion of deceased COPD patients that received PCS followed a similar pattern, overall increasing from 1.2% in 2005 to 16.5% in 2014 (figure 2). The rate of increase over the years appeared to be the same for COPD patients with or without lung cancer (supplementary figure S1).

### Association between PCS and patient characteristics

In the whole COPD cohort, age ≥75 years, being female, cardiovascular comorbidities (myocardial infarction, stroke or heart failure), anxiety, underweight BMI, MRC Dyspnoea score ≥2 and a history of four or more moderate exacerbations or one or more severe exacerbation in the baseline year were significantly associated with an increased odds of receiving PCS after adjusting for all other characteristics (table 2); being overweight or obese was significantly associated with a reduced odds of receiving PCS (table 2). Lung cancer had the strongest association with receiving PCS, even after adjusting for all other characteristics (adjusted OR 14.7, 95% CI 13.5–16).

**TABLE 2:** Association between lung cancer and other patient characteristics and receiving palliative care support for the whole chronic obstructive pulmonary disease (COPD) cohort and the deceased COPD patients

	**Whole cohort**		**Deceased patients**	
	**Crude OR** **(95% CI)**	**Adjusted OR^#^** **(95% CI)**	**Crude OR** **(95% CI)**	**Adjusted OR^#^** **(95% CI)**
**Lung cancer**				
No	1 (reference)		1 (reference)	
Yes	14 (13.0–15.0)***	14.7 (13.5–16.0)***	6.5 (6–7)***	6.1 (5.6–6.6)***
**Age years**				
≤65	1 (reference)		1 (reference)	
65–75	2.0 (1.9–2.1)***	1.6 (1.5–1.7)	0.9 (0.8–1)*	0.9 (0.9–1.1)
≥75	2.4 (2.3–2.6)***	1.9 (1.8–2.1)***	0.6 (0.6–0.7)***	0.7 (0.7–0.8)***
**Sex**				
Female	1 (reference)		1 (reference)	
Male	0.9 (0.8–0.9)***	0.8 (0.8–0.9)***	1.0 (0.9–1.1)	1.0 (0.9–1.1)
**Myocardial infarction**				
No	1 (reference)		1 (reference)	
Yes	1.4 (1.3–1.5)***	1.2 (1.0–1.3)**	0.9 (0.8–0.9)**	0.9 (0.8–1.0)
**Stroke**				
No	1 (reference)		1 (reference)	
Yes	1.4 (1.3–1.5)***	1.1 (1.0–1.3)*	0.8 (0.8–0.9)**	1 (0.8–1.1)
**Heart failure**				
No	1 (reference)		1 (reference)	
Yes	1.6 (1.5–1.8)***	1.5 (1.3–1.6)***	0.7 (0.7–0.8)***	0.9 (0.8–0.9)**
**Anxiety**				
No	1 (reference)		1 (reference)	
Yes	1.2 (1.1–1.2)***	1.3 (1.2–1.4)***	1.3 (1.2–1.4)***	1.2 (1.1–1.3)***
**Depression**				
No	1 (reference)		1 (reference)	
Yes	1.0 (0.9–1.0)***	1.0 (0.9–1.1)	1.2 (1.1–1.3)***	1.1 (1–1.2)
**BMI**				
<19 (underweight)	1 (reference)		1 (reference)	
19–25 (normal)	1.5 (1.4–1.6)***	1.4 (1.3–1.6)***	0.9 (0.8–1)*	0.9 (0.8–1)
25–30 (overweight)	0.7 (0.7–0.8)***	0.7 (0.7–0.8)***	1 (0.9–1.1)	1.0 (0.9–1.1)
≥30 (obese)	0.6 (0.5–0.6)***	0.6 (0.6–0.7)***	0.9 (0.8–1.0)**	0.9 (0.8–1.0)**
**Smoking**				
Ex-smoker	1 (reference)		1 (reference)	
Current smoker	0.9 (0.8–0.9)***	1 (0.9–1.1)	1.1 (1.0–1.2)**	1.0 (0.9–1.0)
**GOLD stage**				
1	1 (reference)		1 (reference)	
2	1.0 (0.9–1.1)		1.2 (1.1–1.4)***	
3	1.4 (1.3–1.5)***		1.0 (0.9–1.1)	
4	2.1 (1.9–2.4)***		1.0 (0.9–1.2)	
**MRC Dyspnoea score**				
1	1 (reference)		1 (reference)	
2	1.3 (1.2–1.5)***	1.2 (1.1–1.4)**	1 (0.8–1.1)	
3	1.9 (1.7–2.2)***	1.6 (1.4–1.8)***	0.8 (0.7–1)*	
4	3.2 (2.8–3.6)***	2.4 (2.1–2.7)***	0.9 (0.7–1)*	
5	7.0 (6.2–7.9)***	5.1 (4.5–5.8)**	1.1 (1–1.3)	
**Exacerbations**				
None	1 (reference)		1 (reference)	
≤2 (moderate)	1.1 (1.1–1.2)***	1.0 (0.9–1.1)	1.0 (1–1.1)	1.0 (1–1.1)
≥3 moderate/≥1 severe	1.6 (1.5–1.6)***	1.2 (1.1–1.3)***	1.0 (0.9–1)	1.0 (1–1.1)

BMI: body mass index; GOLD: Global Initiative for Obstructive Lung Disease; MRC: Medical Research Council. ^#^: adjusted for all other characteristics in the table. *: p<0.05; **: p<0.01; ***: p<0.0001.

In the deceased patients, age ≤65 years, anxiety and not being obese were significantly associated with an increased odds of receiving PCS after adjusting for all other characteristics (table 2). The reduced association of PCS with heart failure may be related to residual confounding from age as it tended towards unity after the multivariate analysis. Lung cancer had the strongest association with receiving PCS, even after adjusting for all other characteristics (adjusted OR 6.5, 95% CI 5.5–6.8).

The sensitivity analysis that included GOLD stage in both multivariate models showed little difference in the odds ratios (whole cohort, lung cancer: adjusted OR 14.4, 95% CI 13–15.9; p<0.0001; deceased patients, lung cancer: adjusted OR 6.1, 95% CI 5.4–6.9; p<0.0001). The sensitivity analysis that included the MRC Dyspnoea score in the deceased patients multivariate model showed little difference in the odds ratios (deceased patients, lung cancer: adjusted OR 6.1, 95% CI 5.5–6.7; p<0.0001).

### Timing of PCS in respect to the patient's death

**FIGURE 3:**
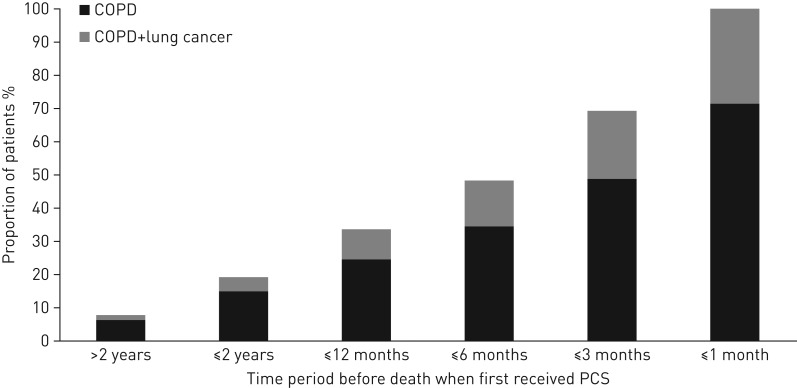
Cumulative proportion of deceased chronic obstructive pulmonary disease (COPD) patients that first received palliative care support (PCS) in relation to the time of their death and the proportion of those that had a lung cancer co-diagnosis.

Only 21.4% of deceased COPD patients received PCS (5595 out of 26 135), of whom 31.5% had a co-diagnosis of lung cancer (1764 out of 5595). Of those that had received PCS, 48.4% received it only within 6 months of their death (of whom 29.1% had lung cancer) (figure 3).

**FIGURE 4:**
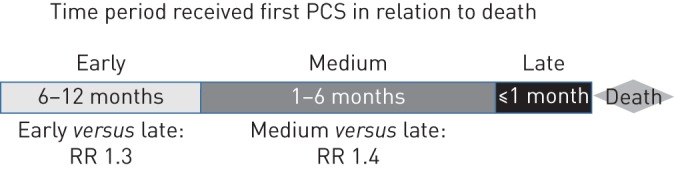
Patients with a dual diagnosis, *i.e.* chronic obstructive pulmonary disease (COPD) and lung cancer, were more likely to receive palliative care support (PCS) earlier before their death than patients with COPD alone. RR: risk ratio (“COPD+lung cancer” *versus* “COPD”). Includes only deceased COPD patients that received PCS within 12 months of their death.

Using multinomial regression, it can be seen that patients with COPD and lung cancer were more likely to receive PCS earlier in their last 12 months of life (≤11 months pre-death) than patients who only had a COPD diagnosis (figure 4). There was a 40% increased chance of receiving support within 1–6 months pre-death and 30% increased chance of receiving PCS within 6–12 months pre-death, compared with receiving palliative care at a late time period (≤1 month pre-death), for patients with both COPD and lung cancer compared with not having lung cancer (middle *versus* late for “COPD+lung cancer” *versus* “COPD alone”: risk ratio 1.4, 95% CI 1.3–1.7; p<0.0001; early *versus* late for “COPD+lung cancer” *versus* “COPD alone”: risk ratio 1.3, 95% CI 1.1–1.6; p<0.01).

## Discussion

From a large primary care cohort of 92 365 COPD patients, only 7.8% of the whole cohort and 21.4% of deceased patients received PCS during the study follow-up. However, just under a third of these patients also had lung cancer; it is highly probable that many patients were receiving PCS in respect of their cancer diagnosis rather than their airways disease. Indeed, only 6% of COPD patients without lung cancer were provided with PCS compared with 50% of the lung cancer patients in the whole cohort. A co-diagnosis of lung cancer had the strongest association with PCS compared with any of the other measured patient characteristics, in both the whole cohort (15 times increased odds of receiving PCS) and in only the deceased patients (6 times increased odds of receiving PCS). Therefore, the largest influence on PCS for COPD patients was not related to the advancement of their airways disease but to having lung cancer. However, it is encouraging that the proportion of COPD patients who received PCS steadily increased between 2005 and 2014, in particular for the deceased COPD patients, both with and without lung cancer. The rise was steepest from 2009 onwards, which could be related to the introduction of the national End of Life Care Strategy, a programme developed to specifically reach patients with nonmalignant life-limiting diseases, and is in keeping with data showing a reduction in undesirable hospital deaths in COPD patients over the same time period [26].

Palliative care is not just synonymous with end-of-life care, but also centres on symptom management, improving a patient's quality of life, and psychological support for the patient and their family. Indeed, modern palliative care approaches are more needs based rather than prognosis based, appropriate for COPD patient's whose life expectancy is difficult to predict [4]. Two other factors, *i.e.* anxiety and MRC Dyspnoea score, indicative of requiring support for psychological and symptom management, respectively, were also significantly associated with PCS in the whole cohort, suggesting PCS was considered for symptom control, but to a far lesser degree than a coexisting diagnosis of lung cancer. Having a high BMI was associated with a reduced chance of receiving PCS; this may reflect a lack of an understanding/willingness to consider PCS in obese COPD patients who may suffer with a relatively increased sensation of breathlessness [27]. Interestingly, older age (≥75 years) was associated with an increased odds of receiving PCS in the whole cohort, but younger age was significantly associated with an increased odds of receiving support in the deceased patients. The deceased patients were a frailer cohort with more comorbidities and more severe clinical features; it could be that in this setting a younger patient is more likely to be considered for PCS than an older patient and it is noteworthy that a similar ageist phenomenon has been recognised in studies addressing referral of general cancer patients to palliative care services [28].

It is also imperative that provision of PCS occurs in a timely manner, not just a few weeks before death [29]. Sadly, this study found that a third of COPD patients only received PCS in the last month of their life. In addition, there was again an inequality between patients with or without a lung cancer diagnosis. Those with lung cancer had an increased chance of receiving PCS earlier in the year before death, whereas patients without lung cancer were much more likely to receive PCS during their final month of life.

The inequality between PCS for COPD patients and lung cancer patients has previously also been noted in other countries [17], yet the two diseases are comparable in their manifestation and severity of symptoms. Moreover, several studies have shown that end-stage COPD patients have at least as many, if not more, physical and psychological needs as patients with end-stage lung cancer [30]. It appears that often it was only the terminal care element of PCS that was provided for COPD patients without lung cancer. However, it has been shown previously that even this aspect of PCS is relatively lacking as COPD patients are more likely to die in hospital than lung cancer patients, which is not in keeping with the general preference to die at home [31, 32].

It has been nearly two decades since a small UK study suggested that PCS was lacking in COPD patients compared with those with lung cancer [13]. Despite an increased recognition during this time that patients with COPD would benefit from a palliative care approach, including implementation of NICE guidelines and the national End of Life Care Programme and Strategy, our data have shown that there has been only a limited increase in PCS uptake. This lack of deliverance from within primary care, including absence of referral to specialist services, has been proposed to be multifactorial and related to difficulties in predicting prognosis, inadequate expertise to engage in palliative care discussions, physician concern in using opioids in chronic respiratory disease, fear of diminishing patients’ hopes and bias against patients with smoking-related lung diseases [27]. Additional strategies are therefore required to help improve access to PCS, including defining and sharing good models of care between the respiratory and palliative care communities, enhanced training in identifying patients who will benefit, communication skills and symptom management, as well as increasing the availability of evidence-based pharmacological and nonpharmacological intervention [4, 33].

### Study limitations

Recording of palliative care provision in primary care is one of the NICE Quality and Outcomes Framework indicators and therefore, fortuitously, this incentive payment programme ensures high recording of PCS. However, it is possible some instances of PCS were not recorded, such as a prescription of opioids/benzodiazepines to palliate breathlessness, but it is likely most GPs would have documented the reason for such prescriptions, especially given they are incentivised to use Quality and Outcomes Framework Read Codes. In this study we only addressed PCS from within primary care, including referrals to specialist services, but not including PCS provided during hospital admissions. Therefore, this study may have excluded some patients who received PCS only through secondary care within the last couple of weeks of their life and were not seen back in primary care after hospital/hospice discharge.

It is very difficult to identify which patients have “end-stage” COPD; therefore, as a proxy, we carried out much of our analysis only on patients who had died. It is possible that we included some patients who did not have end-stage COPD as they may have died from unrelated causes. However, using ONS data we also identified a subgroup of patients whose deaths were highly likely to have been related to their COPD and in that analysis the proportion of patients that received PCS in this subgroup was even lower than for the total deceased population.

There was missing data for the MRC Dyspnoea score and, as we carried out a complete case analysis for the whole cohort, it is possible there was some selection bias as these patients may have had more severe disease (fewer patients had missing MRC Dyspnoea scores in GOLD stage 4 than stages 1–3); however, this should not have affected the findings except to pull the estimate further from unity. Lastly, it is possible that there were other factors (*e.g.* social and cultural preferences) which were not included in our analysis that could have affected our findings; however, due to the strength of the association with lung cancer that relationship is unlikely to have changed considerably.

### Conclusions

This is the first nationally representative study to investigate the provision of PCS for COPD patients. We have shown that the majority of COPD patients within primary care were not provided PCS in their last year of life; when PCS was provided, it appeared to be related to their co-diagnosis of lung cancer rather than their airways disease, including their disease severity (GOLD score and exacerbation frequency) or their perceived respiratory disability (MRC Dyspnoea score). Furthermore, PCS was frequently only given within the last few weeks of life, whereas patients with a lung cancer co-diagnosis were far more likely to receive PCS in a more timely manner.

Many COPD patients suffer from symptom-related distress even when medical treatment has been optimised. The low proportion of patients receiving PCS, not immediately before death, suggests it is an especially poorly provided service with regard to its capacity to reduce symptom burden, improve quality of life and reduce psychological distress. This study advocates an urgent need to improve all aspects of PCS, not just terminal care, for COPD patients within the UK.

## Supplementary material

10.1183/13993003.01879-2017.Supp1 **Please note:** supplementary material is not edited by the Editorial Office, and is uploaded as it has been supplied by the author.
Supplementary table S1 ERJ-01879-2017_Table_S1
Supplementary figure S1 ERJ-01879-2017_Figure_S1

## Disclosures

10.1183/13993003.01879-2017.Supp2 J.K. Quint ERJ-01879-2017_Quint
L. Smeeth ERJ-01879-2017_Smeeth
