# Supplementary material for: Low uptake of palliative care for COPD patients within primary care in the UK
Source: Eur Respir J. 2018 Feb 15;51(2):1701879. doi: 10.1183/13993003.01879-2017 (PMC5898942; doi:10.1183/13993003.01879-2017)
Supplement: Supplementary file 1 [file ERJ-01879-2017_Table_S1.pdf]

Supplementary Table S1. Palliative care Read codes

**Palliative care Read codes**
admission to hospice
admission to hospice for respite
anticipatory palliative care
community specialist palliative care
counselling for end of life issues
current supportive care for terminal illness
discharge by palliative care physician
discharge from hospice
discharge from hospice
discharge from hospice day hospital
discharge from palliative care service
discharge from palliative care service
discharged from community specialist palliative care team
ds 1500 report
end of life care
end of life care pathway
exploring patient's feelings about dying
full care by hospice
gsf advance care plan discussion statement
gsf prognostic indicator stage b (green) - months prognosis
gsf prognostic indicator stage c (yellow) - weeks prognosis
gsf prognostic indicator stage d (red) - days prognosis
gsf supportive care stage 1 - advancing disease
gsf supportive care stage 2 - increasing decline
gsf supportv care stge 3 - last days: cat b - mth prognosis
gsf supportv care stge 3 - last days: cat c - wks prognosis
gsf supportv care stge 3 - last days: cat d - days prognosis
has end of life advance care plan
has end of life care pathway key general practitioner
has end of life care pathway key worker
healthcare prof would not be surprised (gsf surprise qu)
hospice
hospice - nhs
integrated care priorities for end of life
issue of palliative care anticipatory medication box
issue of palliative care just in case box
liverpool care pathway for the dying
notif to primary care oohs of palliative care plan in place
on end of life care register
on gold standards palliative care framework
on liverpool care pathway for the dying
palliative care
palliative care - enhanced services administration

palliative care handover form completed
palliative care plan review
palliative medicine
palliative treatment
patient died in hospice
patient held palliative care record
planned supportive care for terminal illness
preferences relating to death and dying
preferred place of care - hospice
preferred place of death: hospice
prescription of palliative care anticipatory medication
provision of written information about advance care planning
refer for terminal care
refer to terminal care consult
referral to hospice
referral to palliative care physician
referral to palliative care service
referred to community specialist palliative care team
routine admission to hospice
seen by palliative care physician
seen by palliative care service
seen in hospice
shared care - hospice / gp
specialist palliative care
specialist palliative care treatment - daycare
specialist palliative care treatment - inpatient
specialist palliative care treatment - outpatient
terminal care
terminal illness
terminal illness - early stage
terminal illness - late stage
thinking ahead gold standrd advanced care plan disc statemnt
under care of palliative care physician
under care of palliative care physician
under care of palliative care service
under care of palliative care specialist nurse
under the care of community palliative care team
urgent admission to hospice
[v]palliative care
