# Supplementary figures and images for: Low uptake of palliative care for COPD patients within primary care in the UK
Source: Eur Respir J. 2018 Feb 15;51(2):1701879. doi: 10.1183/13993003.01879-2017 (PMC5898942; doi:10.1183/13993003.01879-2017)

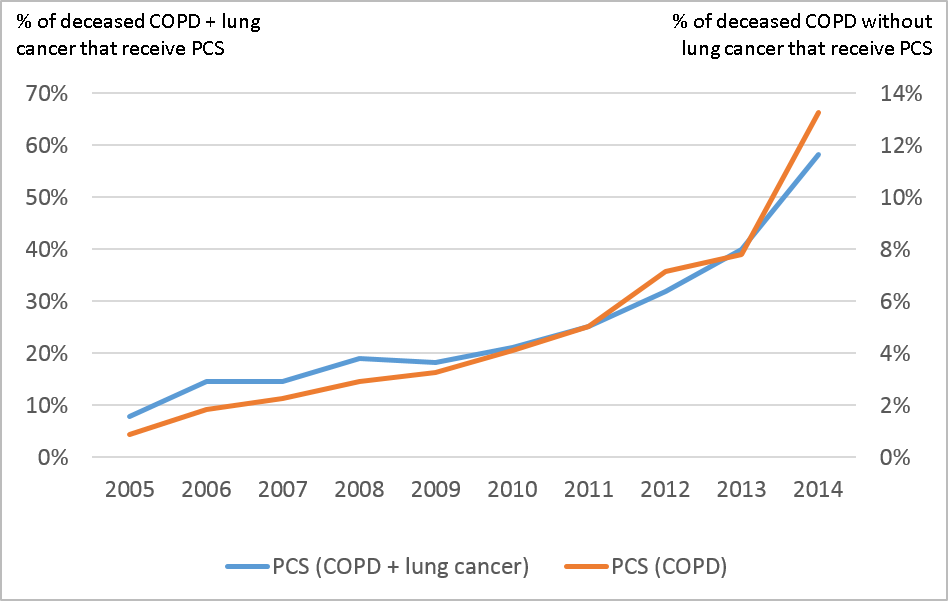

Supplement: Supplementary file 2 [file ERJ-01879-2017_Figure_S1.png]
